# Molecular Tweezers for Biomimetic Recognition of Carbohydrates

**DOI:** 10.1002/cbic.202500123

**Published:** 2025-03-27

**Authors:** Francesco Milanesi, Naufia Mohamedzakaria Shibinasbarveen, Stefano Roelens, Oscar Francesconi

**Affiliations:** ^1^ Department of Chemistry “Ugo Schiff” DICUS Università degli Studi di Firenze Via della Lastruccia 13 SestoFiorentino I‐50019 Firenze Italy; ^2^ Consorzio Interuniversitario Nazionale per la Scienza e Tecnologia dei Materiali (INSTM) 50121 Firenze Italy

**Keywords:** carbohydrates, host‐guest, molecular recognition, molecular tweezers, oligosaccharides

## Abstract

Molecular recognition of biomolecules by means of synthetic receptors is a key topic with several potential applications, which must face the challenge of recognizing relatively large guests in an aqueous medium. Due to their open cavity, molecular tweezers are promising architectures for this purpose that have already shown interesting recognition properties with lipids, nucleic acids, and proteins, thereby eliciting biological activities. Carbohydrates, assembled within glycans, are polar biomolecules of high structural complexity that are particularly difficult to be effectively recognized in water. Notably, in addition to reports on more widely explored structures, significant advances have been achieved exploiting the structural peculiarity of tweezer receptors. In this review a selection of some emblematic examples from the literature of molecular tweezers, cleft and clips targeting carbohydrates is presented, together with a discussion of the advantages and limitations of this type of architecture.

## Graphical abstract

Molecular tweezers have emerged as effective binders for biomolecules such as lipids, nucleic acids, and proteins, eliciting biological activities. Tweezer molecules, which exploit an open and adaptive binding cavity, can target relatively large guests, such as oligosaccharides and glycans, and often provide some advantages over corresponding macrocyclic architectures.

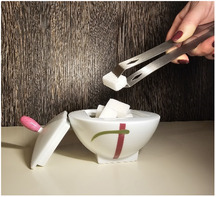

## 1 Introduction

The design and synthesis of receptors for biomometic recognition of biologically relevant guests is an area of growing interest at the edge of supramolecular chemistry and chemical biology.^[^ ^1^, ^2^, ^3^ ^]^ Effective recognition is, however, a major challenge in the design of structures capable of achieving good affinities and selectivities for biological targets for several reasons. In the first place, because the aqueous medium is the biological solvent, the development of biomimetic receptors relying on noncovalent interactions exclusively must overcome the resistance of a medium that is strongly competitive for polar interactions, so that desolvation of both, host and guest, is required for effective recognition.^[^ ^4^, ^5^, ^6^, ^7^, ^8^ ^]^ In the case of carbohydrates, for which the hydration energy is often very high, desolvation is energetically unfavorable, making these ligands particularly hard to bind. This behavior is reflected in the low binding affinities, often in the mm to μm range, that carbohydrates typically show toward proteins, compared to the usually stronger protein–ligand interactions.^[^ ^9^, ^10^ ^]^ Achieving good selectivity with carbohydrates can be a very difficult task as well, because monosaccharides of target glycans most often differ only in the stereochemistry of a single stereogenic centre, making the design of a selective receptor very challenging. In addition, unlike peptides and nucleic acids, carbohydrates can assemble into both linear and branched oligomers via α‐ or β‐ glycosidic linkages, which greatly increases the structural complexity of the guest.^[^ ^11^ ^]^

Despite these difficulties, the primary role of carbohydrates in biological processes^[^ ^12^, ^13^, ^14^ ^]^ has stimulated the endeavor of developing biomimetic receptors that would be effective in the biological medium. Synthetic receptors mimicking carbohydrate–protein interactions, as abiotic alternatives to natural proteins, can be extremely useful in biochemical investigations and in the development of diagnostic and therapeutic strategies.

Different architectures have been explored as synthetic receptors for carbohydrates, including covalent and coordination‐macrocycles,^[^ ^15^, ^16^, ^17^ ^]^ molecular cages,^[^ ^18^, ^19^ ^]^ podand structures,^[^ ^20^, ^21^, ^22^ ^]^ and foldamers,^[^ ^23^, ^24^, ^25^ ^]^ focusing either on rigid preorganized or on flexible adaptive architectures.^[^ ^26^ ^]^ While much effort has been historically devoted to the investigation of molecular recognition of carbohydrates in organic solvents,^[^ ^27^ ^]^ where polar interactions are enhanced facilitating the understanding of the functional and geometric requirements for effective recognition, it is only in more recent years that examples have appeared of synthetic receptors showing affinities and selectivities for saccharides in water comparable to those of natural lectins.^[^ ^15^, ^28^, ^29^, ^30^, ^31^ ^]^ Likewise, while most reports focus on recognition of monosaccharides, appropriately sized receptors have been developed to recognize di‐ and oligosaccharides, which are closer models of more complex structures of glycans, although bringing unavoidable increase in synthetic effort.^[^ ^32^, ^33^ ^]^

**Figure 1:**
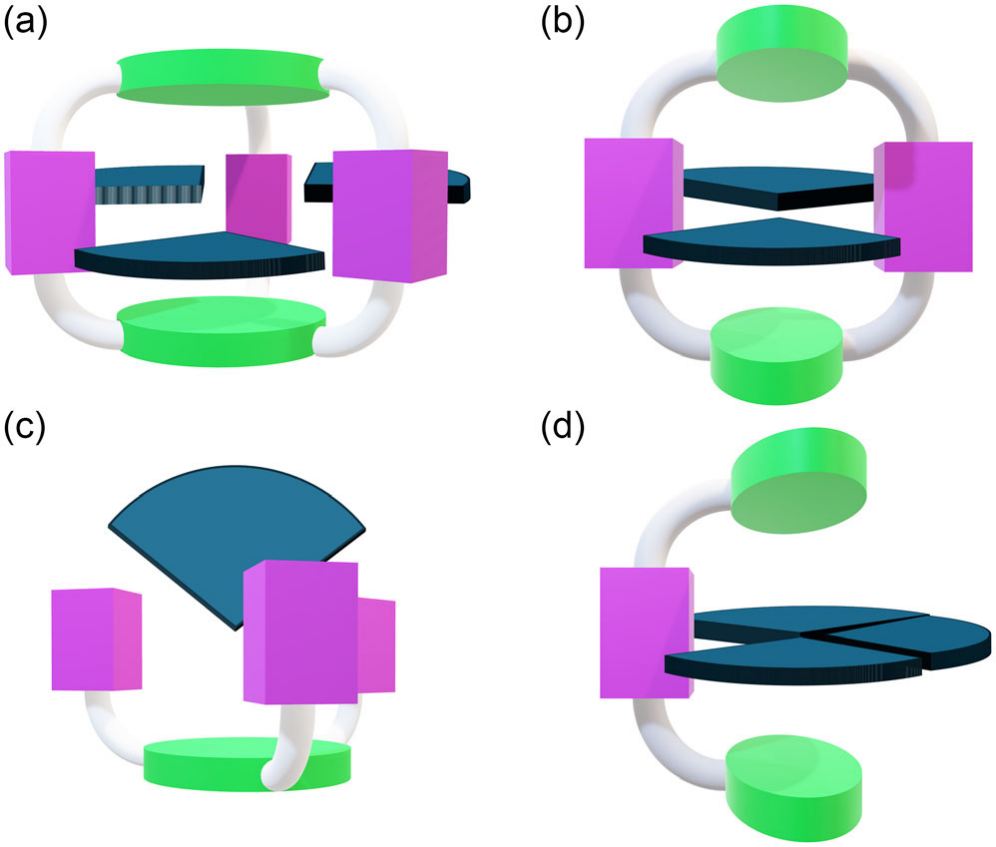
Pictorial illustration of a) cage, b) macrocycle, c) podand, and d) tweezer receptors architectures and their accessibility to the guest (in blue).

**Scheme 1:**
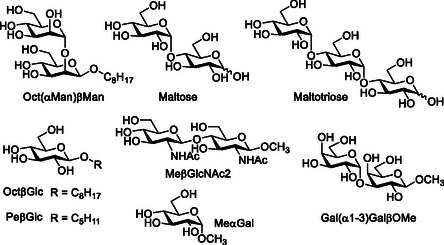
Mono/oligosaccharidic targets and their glycosides investigated for the tweezer receptors overviewed.

In the panorama of receptor architectures, much less explored than the above are structures called molecular tweezers. The latter are characterized by open cavities capable of binding guests through various supramolecular interactions. As first described by Whitlock in 1978,^[^ ^34^ ^]^ molecular tweezers consist of two flat, generally aromatic, identical pincers separated by a moderately rigid tether. The spacer has the dual role of keeping the two pincers apart, thus preventing them from self‐associating, and holding them in a preferential *syn* conformation.^[^ ^35^, ^36^, ^37^, ^38^, ^39^ ^]^ Molecular tweezers have also been called clips or clefts, indicating the previously mentioned structural features.^[^ ^38^ ^]^ In this review, we adopt the definition of tweezer molecules as synthetic receptors characterized by an open cavity freely accessible to the guest on three contiguous sides (**Figure** **1**). As tweezers often possess an acyclic structure, they may be synthesized more easily than macrocyclic architectures. Furthermore, due to the open cavity, tweezers feature a less congested binding site and a more adaptive structure that can accommodate relatively large guests, such as oligosaccharides. Since carbohydrate binding sites of proteins are generally located on the surface, such open cavities provide a bioinspired model for glycan recognition.^[^ ^40^ ^]^ Although molecular tweezers have been successfully used as bioactive agents,^[^ ^41^ ^]^ capable of recognizing biomolecules such as lipids,^[^ ^42^, ^43^ ^]^ proteins,^[^ ^34^, ^38^, ^39^, ^44^ ^]^ and nucleic acids,^[^ ^45^ ^]^ they have only recently been shown to recognize complex oligosaccharides and glycans, proving to be valuable tools to investigate this challenging class of ligands. In this review, an overview of tweezer receptors for carbohydrates will be summarized, focusing on leading structures and carbohydrate binding properties reported in organic solvents as well as in the aqueous medium, together with the investigated carbohydrates (**Scheme** **1**).

## 2 Tweezer Receptors for Carbohydrate Recognition in Organic Solvents

**Scheme 2:**
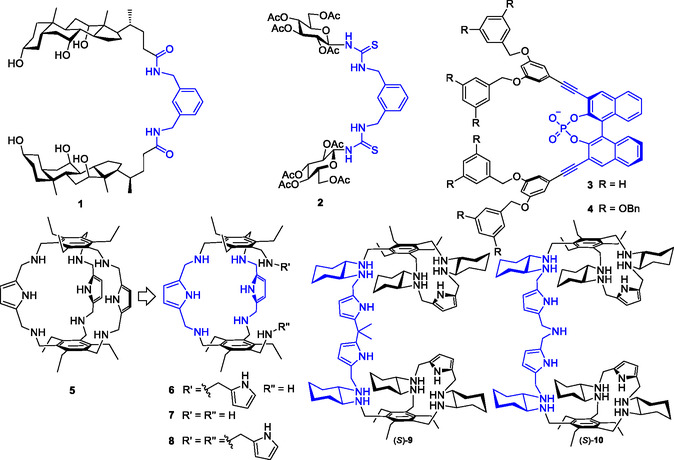
Tweezer‐shaped receptors for molecular recognition of carbohydrates in organic solvents.

In a pioneering stage of the development of synthetic receptors for carbohydrates, which spanned the late ‘80s to the beginning of ‘90s, tweezer receptors were among the first architectures explored, due to their readily synthetical availability. Using a naturally occurring building block, Burrow et al. developed a tweezer receptor by combining two units of cholic acid with a rigid aromatic diamine to prepare the diamide receptor **1** (**Scheme** **2**).^[^ ^46^ ^]^ Steroids are attractive building blocks for supramolecular chemistry because of their size, rigidity, and well‐defined spatial orientation of functional groups. Molecular recognition studies on receptor **1** carried out by ^1^H‐nuclear magnetic resonance (NMR) with n‐pentyl‐β‐D‐glucopyranoside (PeβGlc) in CDCl_3_ showed that the glucoside stabilizes the syn conformation of the receptor, consistent with binding of the carbohydrate between the two cholate moieties. The efficacy of cholic acid as building block in the design of synthetic receptors for carbohydrates was also confirmed by a series of macrocyclic receptors reported by Davis et al. in the same years.^[^ ^47^ ^]^

At the beginning of the new millennium, Mellet and Fernández reported the tweezer receptor **2**, featuring a *m*‐xylylene bis‐thiourea spacer bridging two peracetylated N‐glucopyranosides as pincers.^[^ ^48^ ^]^ The two thiourea moieties act as a strong bidentate hydrogen bonding donor for multipoint interactions with the sugar, leaving the structure with a degree of flexibility to accommodate the guest between the two acetylated hexose units; this binding mode stabilizes the complex through additional interactions between the acetyl groups and the hydroxyl groups of the sugar. Receptor **2** was found to bind the octyl‐β‐D‐glucopyranoside (OctβGlc) with a 1:1 stoichiometry and an association constant of 900 m ^−1^ in CDCl_3_.

In the same years, Diederich et al. taking advantage of the strong anion–diol interactions, developed a series of cleft receptors, such as **3** and **4**, based on a phosphorylated binaphthol, acting both, as a spacer for two Fréchet‐type dendrons, and as a bidentate hydrogen bonding unit.^[^ ^49^ ^]^ The two aromatic pincers provide additional contribution to the binding by establishing Van der Waals and CH–π interactions with the aliphatic backbone of the carbohydrate. Receptors **3** and **4** were found to bind to both α and β octyl glucosides, with a preference for the latter and an affinity for OctβGlc in CDCl_3_ of 1160 and 2280 m ^−1^, respectively. The two receptors were also able to recognize the OctβGlc in a more polar and competitive organic solvent, such as CH_3_CN, with a slightly lower but significant association constant of 350 and 370 m ^−1^, respectively. As for cholic acid receptors, phosphorylated binaphthol receptors were used as building blocks for the synthesis of macrocyclic receptors, which resulted much more effective than the corresponding acyclic receptors.

A reverse strategy was followed by Roelens et al. who converted a cage architecture to a series of cleft receptors.^[^ ^50^ ^]^ Cage receptor **5,** ^[^ ^51^ ^]^ which has a bicyclic architecture leveraging diaminopyrroles as hydrogen bonding pillars and two benzene rings as floor and roof, was found to be very effective in selectively recognizing OctβGlc, with an association constant in CDCl_3_ of 48,300 m ^−1^, corresponding to an affinity of 20.7 μm, but unexpectedly failed to recognize the β glucoside in more polar solvents, such as CH_3_CN. Following the idea that the reason for failure was a binding site too narrow and rigid, a series of cleft receptors were developed by cleaving one of the pillars of the cage, giving rise to an adaptive cavity. Receptors **6**–**8** feature a tweezer's architecture, in which the *syn* conformation is held in place by the macrocyclic structure, with the two benzene rings acting as pincers and additional pyrrole rings as binding hooks. Except for those receptors in which the number of hydrogen bonding groups was heavily decreased compared to the cage receptor **5**, all the other receptors showed a 2‐to‐7‐fold increase in affinity for OctβGlc in chloroform (8.1, 9.2 and 3.5 μm for **6**, **7**, and **8**, respectively). Most important, binding was preserved in more competitive organic solvents, such as chloroform/DMF 70:30, with affinities in the low millimolar range (1.59, 2.01, 0.98 mm for **6**, **7**, and **8**, respectively).

Mazik et al. developed a series of cleft receptors structurally related to **7**,^[^ ^52^ ^]^ featuring alternative hydrogen bonding groups on the scaffold of **7**, with the aim of optimizing the recognition of OctβGlc. The results of the study confirmed diaminopyrrole as the most effective hydrogen bonding motif of the series.

**Figure 2:**
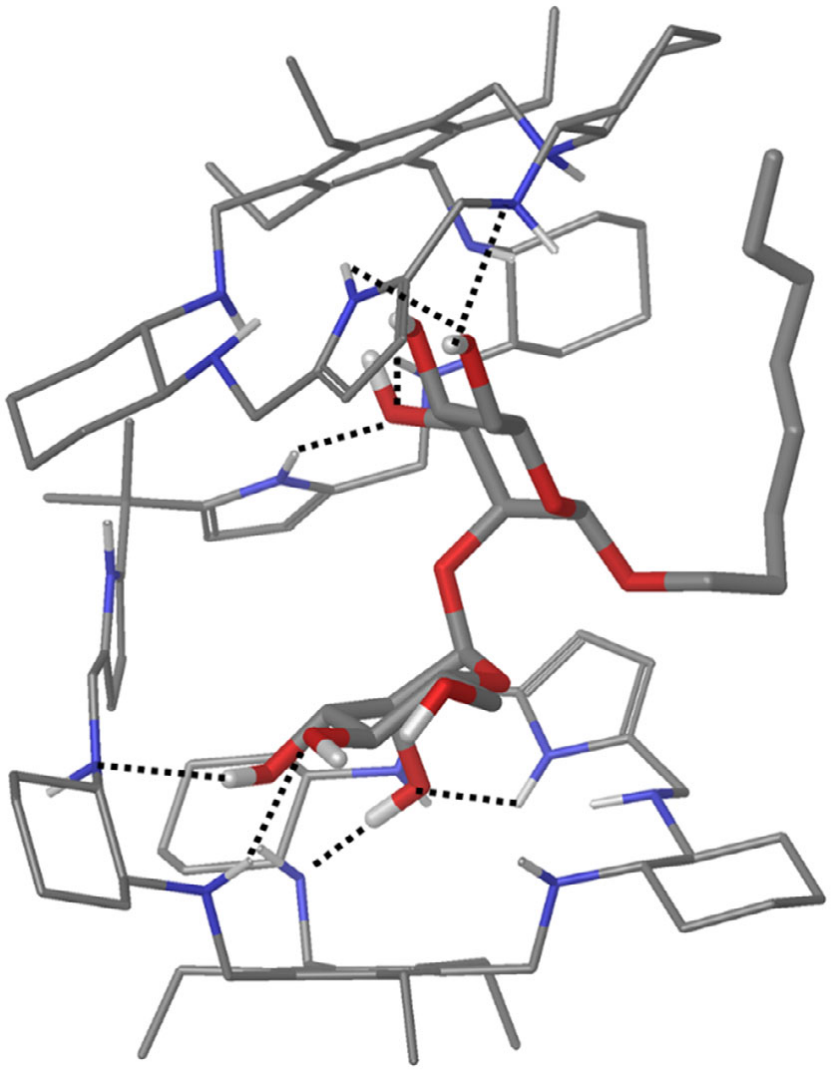
Global minimum structure obtained from a search of the conformational space for the complex between (*S*)‐**9** and Oct(αMan)βMan. Hydrogen bonds are depicted as dashed lines. Adapted with permission.^[^ ^53^ ^]^ Copyright 2013, Wiley‐VCH.

A step forward was made by Roelens et al. with a series of ditopic receptors,^[^ ^53^ ^]^ including, among others, (*S*)‐**9** and (*S*)‐**10**, for recognition of the Manα(1–2)Man dimannoside. This disaccharide is found as terminal unit of high‐mannose type glycans present on the glycoproteins of enveloped viruses, such as HIV, Ebola, Zika, and Corona viruses, whose recognition triggers the infection mechanism; Manα(1–2)Man is also overexpressed in various cancer cells as a consequence of the aberrant misregulation of glycosylation pathways. It must be emphasized that Manα(1–2)Man is a rather challenging guest, due to the relatively large size and the peculiar spatial orientation of the hydroxyl groups. Receptors (*S*)‐**9** and (*S*)‐**10** differ in length and structure of the spacer, which in both cases remains flexible enough to adapt to the dimannoside. The effect of receptor chirality on dimannoside recognition was also investigated, showing for the all‐S enantiomers of two receptors a boost in affinity of almost 2 orders of magnitude with respect to the corresponding monomannosides. Affinities in the low μm range were measured for Oct(αMan)βMan (15 and 64 μm for (*S*)‐**9** and (*S*)‐**10**, respectively) in a highly competitive medium, such as chloroform/DMF 70:30. NMR‐based molecular modeling (**Figure** **2**) of the binding mode of receptor (*S*)‐**9** with Oct(αMan)βMan, revealed the origin of the binding ability of the tweezer receptor, in which the two pincers work in a concerted manner for the recognition of the two mannose units of the dimannoside.

## 3 Tweezer Receptors for Carbohydrate Recognition in Water

**Scheme 3:**
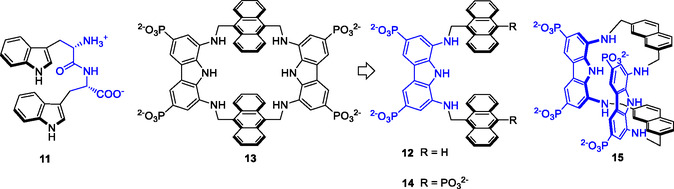
Tweezer‐shaped receptors for molecular recognition of carbohydrates in water.

X‐ray crystallographic structures of sugar–protein complexes inspired Aoyama et al. to develop synthetic receptors for oligosaccharides effective in water.^[^ ^54^ ^]^ Observing that aromatic amino acid side chains, such as the indole of Trp and phenol rings of Tyr, are often found stacking to or even sandwiching with carbohydrate CH moieties, two dipeptide receptors Trp‐Trp (as in **11**, **Scheme** **3**) and Trp‐Tyr were used as molecular pincers for tweezers binding maltose and maltotriose. Tweezer receptor **11** exhibited association constants in water of 1 and 8 m ^−1^ for maltose and maltotriose, respectively, demonstrating the efficacy of the tweezer architecture and the primary role of CH–π interactions in the molecular recognition of carbohydrates in water.

Curiously, after Aoyama's pioneering example, in the design of receptors for molecular recognition of carbohydrates in water the concept of molecular tweezers was not followed for a long time, whereas other types of architectures, such as macrocyclic, cage, and temple structures were preferentially explored for the scope. More recently, the tweezer architecture has been successfully rediscovered, whereby particularly interesting results were obtained on the recognition of complex guests, such as oligosaccharides and glycans.

While the recognition of relatively large guests, such as oligosaccharides, is generally approached with more complex receptors, the opposite approach was followed by Francesconi et al. reporting in 2021 a simple acyclic receptor **12**, which turned out to be particularly effective and selective in the recognition of N,N’‐diacetylchitobiose, a.k.a. GlcNAc_2_, which is biologically relevant because is part of the core structure of all N‐glycans in eukaryotes.^[^ ^55^ ^]^ The tweezer receptor, consisting of two anthracene rings as pincers and a diaminocarbazole as spacer, originates from the molecular simplification of the previously reported macrocyclic receptor **13**,^[^ ^56^ ^]^ which showed steric hindrance in the selective recognition of 1,4‐linked disaccharides, such as cellobiose, maltose, lactose, and GlcNAc_2_. Removal of one diaminocarbazole unit from **13** relieved the constraint imposed by the macrocyclic structure, providing an open cavity capable of accommodating disaccharides. The tweezer receptor retained the anthracene and diaminocarbazole (a rigid alternative to the previously employed diaminopyrrole unit)^[^ ^57^ ^]^ binding elements of the parent macrocycle. Receptor **12** showed the ability to selectively recognize MeβGlcNAc_2_ in water among other 1,4‐linked disaccharides, with a remarkable affinity of 160 μM, overriding by one order of magnitude the affinity of the pseudo‐lectin hevein from *Hevea* *brasiliensis* for the same disaccharide.

**Figure 3:**
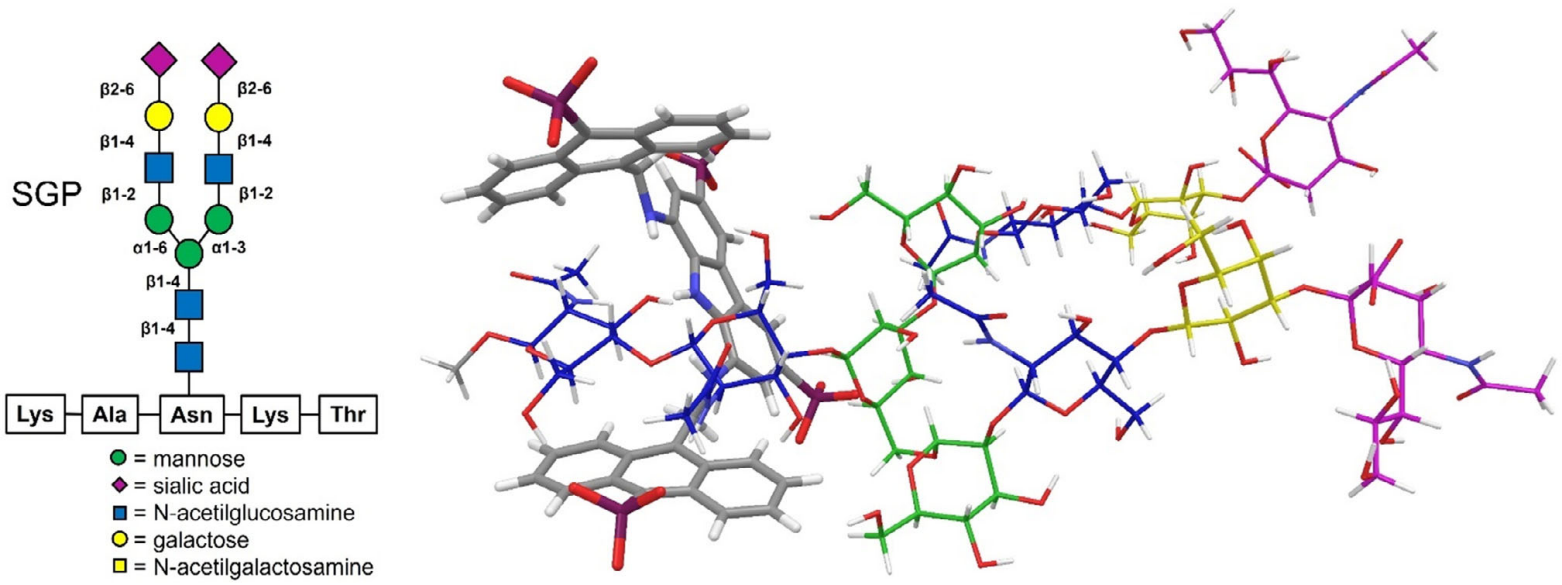
Schematic structure of the sialogycopeptide (SGP). NMR‐based molecular modeling of the binding mode between **14** and the glycoside of SGP. Adapted with permission.^[^ ^58^ ^]^ Copyright 2023.

Since the GlcNAc_2_ disaccharide, which is directly linked to an asparagine residue of proteins, is located at the stem of N‐glycans, the tweezer architecture has recently been used to intercept the GlcNAc_2_ disaccharide in the core structure of the sialoglycopeptide (SGP).^[^ ^58^ ^]^ The tetraphosphonate analog **14**, designed to circumvent the self‐association phenomenon of the progenitor, showed the same affinity for SGP as that measured for the GlcNAc_2_ disaccharide. Despite the undecasaccharide structure and the pentapeptide chain of SGP, the recognition occurs exclusively with the GlcNAc_2_ unit, as confirmed by NMR studies and molecular modeling calculations (**Figure** **3**).

In a recent study by Francesconi et al. the potential of the tweezer architecture for the recognition of oligosaccharides was further demonstrated with receptor **15**.^[^ ^59^ ^]^ Receptor **15** combines in a unique structure the tweezer conformation of receptor **12** with a macrocylic architecture analogous to receptors **6**–**8**, which pre‐organizes the aromatic pincers in a syn conformation and increases the hydrogen bonding interaction with the guest with respect to receptor **12** by means of two diaminocarbazole units. Due to the latter groups, receptor **15** selectively binds the axially substituted methyl‐α‐D‐galactopyranoside (MeαGal), showing an affinity of 4.4 mm. Interestingly, the open cavity of the molecular tweezers also allows **15** to recognize Gal(α1‐3)GalβOMe with an affinity of 3.97 mm. This disaccharide is structurally related to the α‐Gal epitope [Gal(α1‐3)Gal(β1‐4)GlcNAc],^[^ ^60^ ^]^ which is largely expressed in non‐primate mammals but absent in humans that produce specific IgE, thus hampering xenotransplantation and causing the alpha‐gal syndrome, a meat allergy.

## 4 Summary and Outlook

Carbohydrates are ubiquitous biomolecules that play key roles in biological systems, many of which have yet to be elucidated. Molecular recognition by means of synthetic receptors has the potential to shed light on the molecular basis of carbohydrate recognition, and to modulate carbohydrate‐mediated processes, thereby facilitating further development in biological applications. However, carbohydrates, especially when assembled into oligomers, are challenging guests due to their structural complexity and the strong interactions with the aqueous medium. In the development of synthetic receptors, molecular tweezers have demonstrated their efficacy to recognize large biomolecules, such as proteins, lipids and nucleic acids, although their implementation in carbohydrate recognition has been scarcely explored with respect to other architectures. Nevertheless, an increasing interest on tweezer structures has recently appeared in the literature, revitalizing the subject and reporting promising results. Although studies on the biological activity of tweezers receptors for carbohydrates have not yet been reported in the current literature, an increasing interest in exploring their biological potential can easily be foreseen in the near future.

Synthetically accessible acyclic molecular tweezers were among the first architectures investigated for carbohydrate recognition in organic solvents. However, in many cases they were used as input for the design of the corresponding macrocyclic receptors. On the contrary, an approach aimed at simplifying cage or macrocyclic receptors to obtain tweezer structures has only recently been adopted, bringing significant results, not only in organic solvents but also in water. Recognition of complex oligosaccharides and N‐glycans was achieved with high affinities and selectivities, comparable to those shown by natural lectins. Results demonstrate that molecular tweezers are promising architectures for further development of receptors for carbohydrates.

## Conflict of Interest

The authors declare no conflict of interest.
